# An antiretroviral drug-naïve human immunodeficiency virus-1 infected woman with a persistent non-reactive proviral deoxyribonucleic acid polymerase chain reaction: a case report

**DOI:** 10.1186/1752-1947-7-152

**Published:** 2013-06-10

**Authors:** Anfumbom KW Kfutwah, Valerie Ngono, Paul Alain Ngoupo, Richard Njouom

**Affiliations:** 1 Service de Virologie, Centre Pasteur du Cameroun, BP 1274 Yaoundé, Cameroun; 2 Department of Biochemistry, University of Yaounde 1, Yaoundé, Cameroon; 3 Faculty of Science, Université de Rouen, 76 183 Rouen, France

## Abstract

**Introduction:**

Replication of the human immunodeficiency virus involves an obligatory step of reverse transcription of the viral ribonucleic acid genome into a double-stranded deoxyribonucleic acid, and subsequent integration of the deoxyribonucleic acid into the human chromatin to form the proviral deoxyribonucleic acid. This proviral human immunodeficiency virus deoxyribonucleic acid is a critical marker for the diagnosis of acute infections, mother-to-child transmissions and for the confirmation of indeterminate serological reactions. We describe a case of a human immunodeficiency virus positive woman, naïve to antiretroviral treatment, who was persistently negative for human immunodeficiency virus proviral deoxyribonucleic acid polymerase chain reaction. This observation, to the best of our knowledge, is the first time that it has been described in Africa.

**Case presentation:**

A 28-year-old Gabonese woman living in Cameroon requested a human immunodeficiency virus diagnosis in our laboratory. She had an unprotected heterosexual contact 6 months earlier while on vacation in Gabon. The request for a human immunodeficiency virus test was as a result of apprehensions developed after the exposure episode. Human immunodeficiency virus serological examinations were ambiguous and confirmatory tests (including human immunodeficiency virus proviral deoxyribonucleic acid polymerase chain reaction) were carried out. Apart from the human immunodeficiency virus proviral deoxyribonucleic acid polymerase chain reaction that was persistently negative, all other polymerase chain reactions carried out were positive. The deoxyribonucleic acid sequences have been submitted to the GenBank database with accession numbers: KC626022, KC626023 and KC626024 for the protease, reverse transcriptase and gp41 genes respectively.

**Conclusion:**

The persistently negative human immunodeficiency virus proviral deoxyribonucleic acid polymerase chain reaction in a person with a confirmed human immunodeficiency virus infection is of immense importance in the human immunodeficiency virus diagnostic field. This could highlight the fact that cases of false negative human immunodeficiency virus proviral deoxyribonucleic acid polymerase chain reactions exist especially with the high genetic variations observed with human immunodeficiency virus. The challenges presented by such false negative tests in the identification of acute infections, mother-to-child transmissions and the confirmation of indeterminate serological reactions are daunting. These data therefore would be invaluable especially to clinicians in Africa where non-B human immunodeficiency virus subtypes circulate.

## Introduction

Proviral human immunodeficiency virus (HIV) deoxyribonucleic acid (DNA) is a useful marker for the diagnosis of acute infections, resolution of indeterminate HIV serological tests as well as the diagnosis of neonates born to HIV seropositive mothers. In infancy and up to the age of 18 months, placentally transferred HIV antibody from the mother precludes antibody testing for diagnosis. In Cameroon, the importance of proviral HIV DNA polymerase chain reaction (PCR) in the diagnosis of early infant infections has been shown [1]. Serological indeterminate cases in Cameroon have been largely attributed to the elevated number of false positive reactions observed. HIV indeterminate cases in our laboratory are defined as those samples that show discordant results between the first two enzyme immune assays (EIA) or between the EIAs and serotyping for HIV group discrimination. In our laboratory, HIV confirmations of serological indeterminate cases are a routine practice using proviral DNA PCR (Generic HIV DNA Cell, Biocentric, Bandol, France). This technique has been used to confirm HIV infections in children on antiretroviral (ARV) therapy who showed HIV indeterminate or negative serological profiles after 18 months [2].

In this case report, we describe a serological indeterminate HIV positive woman with a repeatedly negative proviral DNA PCR that was confirmed using alternate assays. The implications of such false negative HIV proviral DNA in an area that has recently witnessed an upsurge in the use of proviral DNA PCR especially for HIV diagnosis in neonates and confirmation of HIV serologically indeterminate cases is of immense importance to public health.

## Case presentation

A 28-year-old Gabonese woman living in Cameroon requested an HIV diagnosis in our laboratory. She reported an unprotected heterosexual contact 6 months earlier while on vacation in Gabon. Repeated serological analyses as previously described [3] with slight modifications were carried out. Briefly, the initial serological analyses comprised two fourth-generation EIAs (Murex Ag-Ab combination Abbott and Genscreen ULTRA Ag-Ab Bio-Rad, Marnes-la-Coquette, France), followed by an in-house serotyping assay for HIV group discrimination [3,4]. With the first sample collected (D0) we observed discordant serological results between the different screening assays used. A fresh sample (D15) was collected 2 weeks later and similar serological results were obtained. Further serological and molecular tests were therefore carried out with D15 in order to confirm the HIV status of the woman. A final sample (D49) for confirmation of these serological and molecular tests was collected 6 weeks after the first sample. The molecular tests made use of the following genetic material extracted from samples: ribonucleic acid (RNA) was extracted from 200μL of plasma using QIAamp® Viral RNA mini kit (Qiagen, Courtaboeuf, France) and DNA was extracted from 200μL of buffy coat using QIAamp® DNA mini kit (Qiagen) according to the manufacturer’s recommendations. In all, the following supplementary tests were carried out:

1. HIV Western blot (Bio-Rad).

2. HIV-1 group M RNA viral load (Biocentric) which targets the long terminal repeat (LTR) gene of HIV [5].

3. Proviral HIV-1 group M DNA PCR (Biocentric) which targets the LTR gene of HIV [6].

4. An “in-house” proviral HIV-1 group O PCR for the detection of proviral DNA (LTR gene).

5. An “in-house” plasma RNA viral load for HIV-1 group O (integrase gene).

6. A “group O and M-specific PCR” after a reverse transcription step from RNA on the HIV integrase gene, as described by Heyndrickx and colleagues [7].

7. Specific PCRs for the following genes: protease, reverse transcriptase [8] and gp41 [9] for HIV-1 group M. Similar HIV-1 group O-specific PCRs were carried out for the same regions as we previously described [3].

As shown on Table 1, Western blotting on the sample collected at D15 revealed the presence of the following peptides: gp160, trace gp110/120 and gp41, p68, p55, p40, p25 and p18. CD4 T-lymphocyte cell count using the FACSCount™ (Becton Dickinson) was 893 cells/mm^3^ at D49. HIV-1 group M RNA viral load was 3.9 log copies/mL at D15 and 3.3 log copies/mL at D49. By contrast, proviral HIV-1 DNA group M and O (“in-house”) PCRs were repeatedly undetectable at D15 and D49 (repeated three times consecutively for each time point). Non-specific primers (Table 2) used to amplify the integrase genes of HIV-1 group M were detectable. Furthermore, Real-Time (RT) RNA PCRs amplified the reverse transcriptase, protease and gp41 genes of HIV-1 group M but not of HIV-1 group O indicating therefore an HIV-1 group M infection. Primers used for these tests are presented in Table 2. Partial sequencing of the amplified products and phylogenetic analysis of the reverse transcriptase, protease and gp41 genes further revealed an infection with an HIV-1 group M subtype A virus. The DNA sequences of these genes have been submitted to the GenBank database with the following accession numbers: protease – KC626022, reverse transcriptase – KC626023 and gp41 – KC626024. In addition to the above analyses, we did not find any major or minor amino acid mutations that could confer resistance to drugs of the non-nucleotide reverse transcriptase inhibitors and those of the nucleotide reverse transcriptase inhibitor groups following the Agence Nationale de Recherches sur le Sida et les Hépatites Virales 2012 Algorithm [10]. However, amino acid analyses in the protease gene showed amino acid mutations at codon 63 (L63P) which is associated with a polymorphism at a drug resistant site to lopinavir. H69K and L89M were also present in the protease gene which confer a possible resistance to tipranavir for the subtype B strain but there is insufficient data for non-B subtypes. These results suggest that this virus strain present in the patient could be susceptible to the majority of ARVs currently used in Cameroon.

**Table 1:** Different serological and molecular tests carried out on three different samples collected from the same patient at different dates

**Sample code (interval of sample collection)**	**4th generation EIAs**	**Serotyping**	**Western blot**	**Proviral DNA PCR group M (Biocentric)**	**Proviral DNA PCR group O (“in-house”)**	**Viral load RNA M (Biocentric)**	**Non-specific PCR (integrase gene)**	**PCR RT, protease and gp41 genes of HIV-1 group M**	**PCR RT, protease and gp41 genes of HIV-1 group O**
**D0 (first sample)**	Positive	Not detected	/	/	/	/	/	/	/
**D15 (2 weeks)**	Positive	Not detected	gp160, gp110/120 (trace), gp41 (trace), p68, p55, p40, p25, p18	Negative*	Negative*	3.9 log/mL	Positive*	Positive*	Negative*
**D49 (6 weeks)**	Positive	Not detected	/	Negative*	Negative*	3.3 log/mL	Positive*	Positive*	Negative*

EIA, enzyme immune assay; HIV, human immunodeficiency virus; PCR, polymerase chain reaction; RNA, ribonucleic acid; RT, reverse transcriptase; *tested three times consecutively; / test not carried out.

**Table 2:** Sequences of primers used for alternate polymerase chain reactions

**Genome region**	**Primer name**	**Sequence (5´ to 3´)**	**Reference**
**Group M** ***pol***			
Protease (RT PCR)	Outer sense : 5´Prot1	TAATTTTTTAGGGAAGATCTGGCCTTCC	Ref. 8
	Outer antisense : 3´Prot1	GCAAATACTGGAGTATTGTATGGATTTTCAGG	Ref. 8
(nested PCR)	Inner sense : 5´Prot2	TCAGAGCAGACCAGAGCCAACAGCCCCA	Ref. 8
	Inner antisense : 3´Prot2	AATGCTTTTATTTTTTCTTCTGTCAATGGC	Ref. 8
Reverse transcriptase (RT PCR)	Outer sense : MJ3	AGTAGGACCTACACCTGTCA	Ref. 8
	Outer antisense : MJ4	CTGTTAGTGCTTTGGTTCCTCT	Ref. 8
(nested PCR)	Inner sense : A35	TTGGTTGCACTTTAAATTTTCCCATTAGTCCTATT	Ref. 8
	Inner antisense : NE135	CCTACTAACTTCTGTATGTCATTGACAGTCCAGCT	Ref. 8
**Group M** ***env***			
Gp41 (RT PCR)	Outer sense : 1S	TGGAGGAGGAGATATGAGG	
	Outer antisense : AS1	GTGAGTATCCCTGCCTAACTCTAT	Ref. 9
(nested PCR)	Inner sense : T20S1	GAGGGACAATTGGAGAAGTGAATT	Ref. 9
	Inner antisense : 2AS	CTACCAAGCCTCCTACTATC	Ref. 9
**Group O** ***pol***			
Pol (RT PCR)	Outer sense : ProtO	TTCAAYTGTGGRAAAGAGGGAC	Ref. 3
	Outer antisense : PolOL1	CTAATTCCTTGATAGATTTGACT	Ref. 3
*Protease (nested PCR)	Inner sense : Prot4	CAGCCCCACCRATGGAGG	Ref. 3
	Inner antisense : NouvL	CATTGTTTTACTTTTGGTCCAT	Ref. 3
*Reverse transcriptase (nested PCR)	Inner sense : PolOF1	CAGTATTRGTGGGACCTACTCCTGTT	Ref. 3
	Inner antisense : PolOL2	GGCTGTACTGTCCAYTTGTCTG	Ref. 3
**Group O** ***env***			
Gp41 (RT PCR)	Outer sense : V3DURA	ATTCCAATACACTATTGTGCTCCA	Ref. 3
	Outer antisense : gp41NE3	TAAGTTGCTCAAGAGGTGGTA	Ref. 3
(nested PCR)	Inner sense : OFU	TAAAACCTTTTAGTGTRGCAC	Ref. 3
	Inner antisense : 8393L	GTTGATATCCCTGCCTAATG	Ref. 3
Group O and M-specific PCR (integrase region)	Outer sense : 4235	CCCTACAATCCCCAAAGTCAAGG	Ref. 7
	Outer antisense : 4538	TACTGCCCCTTCACCTTTCCA	Ref. 7
(nested PCR)	Inner sense : 4327	TAAGACAGCAGTACAAATGGCAG	Ref. 7
	Inner antisense : 4481	GCTGTCCCTGTAATAAACCCG	Ref. 7

*PCR* polymerase chain reaction, *Ref.* Reference, *RT* reverse transcriptase, * use the same RT PCR primers from the pol gene.

## Discussion

To the best of our knowledge, only two false negative HIV-1 proviral DNA PCRs have been elaborately described. The first case ensued from an attempt to diagnose mother-to-child transmission of HIV in an 8-month-old female baby of Sudanese origin in the United States of America (USA) [11] and the second from a treatment-naïve patient with primary infection in Thailand [12]. The explanation of the false negativity in Thailand was based on mutational changes because amplification using modified primers and thermal cycling parameters resulted in all specimens testing positive. In a recent study, Weidner and colleagues [13] showed that four of 80 samples could not be amplified in a proviral DNA PCR but this was due to a low DNA content of <8ng/μL of the specimens. This same study also showed a specificity of 100% for the 20 negative samples tested [13]. However, several cases of false positivity have been reported [14,15]. In fact Busch *et al.* have reported a very high rate of 18.5% false positive HIV DNA PCR in uninfected individuals [14].

We describe an HIV positive case that was repeatedly negative over two time points when tested for HIV proviral DNA up to 7 months after an unprotected heterosexual contact. Initial serological analyses were not conclusive and such indeterminate cases in our setting are routinely confirmed by an HIV DNA PCR. Although the amounts of DNA were not measured after extraction to ascertain whether DNA levels were low, we simultaneously ran known HIV positive and negative samples as controls to ensure the validity of our HIV proviral DNA PCRs. These controls are standardized samples that were collected from known HIV positive and negative patients in our laboratory and are routinely used as internal controls. In all PCRs the internal controls were validated and the test samples were consecutively negative. Considering that the HIV proviral DNA PCR was negative in this woman who had indicated an unprotected sexual exposure combined with an indeterminate serology, other complementary tests were carried out. These tests revealed an HIV-1 group M subtype A infection. This subtype has been shown to circulate in the central African region, including Gabon and Cameroon, in addition to the predominating circulating recombinant form (CRF)02_AG [16]. A previous study had failed to diagnose HIV subtype C in a baby in the USA [11]. Cunningham and colleagues made a similar observation with subtype E CRF A/E in Thailand [12] which suggests that occasional mutations especially with non-B subtypes could result in false negative proviral DNA tests or sub-optimal amplifications. In this study the proviral DNA was tested by a PCR that targets a segment of the highly conserved HIV-1 LTR. The primers and probe have earlier been evaluated and optimized for HIV RNA quantification of diverse viral strains (including non-B subtypes) originating from several countries. These modified primers and probe therefore allowed for the efficient RNA quantification of samples from the central African subregion (Cameroon and the Central African Republic) [5].

Cunningham and colleagues further showed that inclusion of modified primer pairs could alter these false negative results [12]. Some primer pairs therefore could lead to sub-optimal amplifications and hence low DNA levels. Very low DNA levels resulting probably from these sub-optimal amplifications have been shown to be one of the causes of false negative results of HIV DNA PCR [13]. Our primers and probe have already been shown to be effective in the central African region where essentially non-B HIV subtypes including subtype A circulates. Although this study did not verify oligonucleotide primer mismatch by altering the primers used, it could be important to note here that HIV DNA PCR coupled with the above mentioned alternate assays have been in routine use in our laboratory since 2006 and this is the first case of a false negative HIV DNA PCR that has been noted. This occurrence therefore is relatively rare and could be a spontaneous case of a sub-optimal amplification that is strain specific. Since we could not sequence the LTR gene, the proviral DNA false negativity observed in this study therefore does not exclude the presence of unusual polymorphisms, insertions or deletions that could be present in the LTR. Low plasma viral loads of 3.9 log copies/mL at D15 and 3.3 log copies/mL at D49 (less than 10,000 copies/mL) targeting the same gene (LTR) almost certainly agree with the fact that we could not detect proviral DNA because of these alterations. However, it could be argued that these low viral loads corroborate with the high CD4 cell counts of 893 cells/mm^3^ observed at D49. This could therefore suggest a recent infection with the characteristic low viral load high CD4 cell counts profile.

## Conclusion

In our setting, HIV-1 proviral DNA PCR is routinely used for the confirmation of HIV serological indeterminate cases and the early diagnosis of HIV in neonates. It could be deduced from this study that a negative HIV-1 proviral DNA PCR result indicates only the absence of HIV-1 proviral DNA in the specimen tested and does not exclude a diagnosis of the disease. Negative results should therefore be interpreted with caution, considering the patient's risk factors for HIV infection. The need for good communication between the laboratory and the clinician cannot be over emphasized especially in areas where non-B subtypes predominate or in situations where infection with non-B subtypes is suspected. Although the occurrence of HIV-1 proviral DNA negativity in treatment-naïve patients is quite rare (this is the third elaborate report since 2002), follow-up testing with complementary tests is recommended for high-risk patients with initially HIV-1 proviral DNA negative test results. Another solution could be to optimize and validate existing HIV proviral DNA PCRs with non-B HIV subtypes.

## Consent

Written informed consent was obtained from the patient for publication of this case report and any accompanying images. A copy of the written consent is available for review by the Editor-in-Chief of this journal.

## Abbreviations

ARV: Antiretroviral; CRF: Circulating recombinant form; EIA: Enzyme immune assay; HIV: Human immunodeficiency virus; LTR: Long terminal repeat; PCR: Polymerase chain reaction; RNA: Ribonucleic acid; USA: United States of America.

## Competing interests

The authors declare that they have no competing interests.

## Authors’ contributions

AKWK and RN drafted the manuscript. VN and PAN carried out the analyses. AKWK, PAN, VN and RN interpreted the analyses, reviewed the manuscript and approved the final version. All authors read and approved the final manuscript.
